# Systematic Mutational Analysis of the Putative Hydrolase PqsE: Toward a Deeper Molecular Understanding of Virulence Acquisition in *Pseudomonas aeruginosa*

**DOI:** 10.1371/journal.pone.0073727

**Published:** 2013-09-10

**Authors:** Benjamin Folch, Eric Déziel, Nicolas Doucet

**Affiliations:** 1 INRS-Institut Armand-Frappier, Université du Québec, Laval, Québec, Canada; 2 PROTEO, The Québec Network for Research on Protein Function, Structure, and Engineering, Université Laval, Québec, Québec, Canada; 3 GRASP, Groupe de Recherche Axé sur la Structure des Protéines, McGill University, Montréal, Québec, Canada; The Scripps Research Institute and Sorrento Therapeutics, Inc., United States of America

## Abstract

*Pseudomonas aeruginosa* is an important opportunistic human pathogen that can establish bacterial communication by synchronizing the behavior of individual cells in a molecular phenomenon known as “*quorum sensing*”. Through an elusive mechanism involving gene products of the *pqs* operon, the PqsE enzyme is absolutely required for the synthesis of extracellular phenazines, including the toxic blue pigment pyocyanin, effectively allowing cells to achieve full-fledged virulence. Despite several functional and structural attempts at deciphering the role of this relevant enzymatic drug target, no molecular function has yet been ascribed to PqsE. In the present study, we report a series of alanine scanning experiments aimed at altering the biological function of PqsE, allowing us to uncover key amino acid positions involved in the molecular function of this enzyme. We use sequence analysis and structural overlays with members of homologous folds to pinpoint critical positions located in the vicinity of the ligand binding cleft and surrounding environment, revealing the importance of a unique C-terminal α-helical motif in the molecular function of PqsE. Our results suggest that the active site of the enzyme involves residues that extend further into the hydrophobic core of the protein, advocating for a lid-like movement of the two terminal helices. This information should help design virtual libraries of PqsE inhibitors, providing means to counter *P. aeruginosa* virulence acquisition and helping to reduce nosocomial infections.

## Introduction

*Pseudomonas aeruginosa* is a prevalent opportunistic pathogen that can trigger serious infections in a wide variety of hosts, including plants, insects, and animals [1]. In humans, *P. aeruginosa* is a major nosocomial pathogen responsible for many hospital and clinical outbreaks worldwide [2]. It is also responsible for persistent drug refractile lung infections [3], sepsis in burn patients [4], as well as morbidity and mortality among individuals suffering from cystic fibrosis [5], [6]. This pathogen is difficult to eradicate due to its high level of antibiotic resistance, which involves a variety of molecular mechanisms including multidrug efflux pumps, outermembrane porins, and inactivating enzymes [7]. From a molecular perspective, *P. aeruginosa* infections are linked to the synthesis of various virulence factors such as proteases, rhamnolipids, hydrogen cyanide, exotoxins, and phenazines, which cause a number of inflammatory and oxidative stresses leading to dysfunction of the respiratory tract [7]. To efficiently regulate the expression of most of its virulence factors, *P. aeruginosa* employs a quorum sensing regulatory network comprising at least three cell-to-cell signaling systems [8], [9]: two acyl-homoserine lactone (AHL)-based LuxRI quorum-sensing systems and the MvfR (PqsR) system functioning through 2-alkyl-4 (1*H*)-quinolones (AQs) (such as the *Pseudomonas* Quinolone Signal-PQS).

Previous studies have shown that disruption of the AQ system leads to decreased *P. aeruginosa* virulence and altered cell-to-cell communication [10]–[16]. Enzymes encoded by the *pqsABCDE* operon are required for the synthesis of AQs. Interestingly, the *pqsE* gene is not involved in AQ production [10], [12]{Déziel, 2004 #9;Déziel, 2004 #2128;Gallagher, 2002 #1995} but still represents a key factor for the full-fledged virulence acquisition of *P. aeruginosa* [11], [17]. Indeed, the encoded PqsE enzyme is mandatory for the regulation of essentially all genes controlled by quorum sensing through AQs [18]. As such, the biological function played by PqsE leads to the synthesis of a number of virulence factors, predominantly pyocyanin, a chromogenic phenazine essential for the acquisition of the virulence phenotype. Despite considerable research efforts devoted to PqsE over the past few years, the biological function of this 34 kDa putative hydrolase that adopts the typical three-dimensional structure of the metallo-hydrolases superfamily still remains elusive [17]–[20]. To date, the natural substrate (s) of PqsE is (are) still unknown and the regulatory role (s) played by the enzyme is (are) still debated. To provide further insight on the molecular function and catalytic mechanism of this putative hydrolase, we carried out a structural investigation and alanine scanning of the PqsE active site by site-directed mutagenesis. Through the *in vivo* enzymatic quantification of PqsE variants complementing a pyocyanin-deficient *P. aeruginosa pqsE^−^* mutant strain, we identified residues playing key roles in the biological function of this regulatory enzyme and quantified their stability upon mutation using the PoPMuSiC software [21]. We also present further insights on the functional importance of two structural motifs unique to this representative member of the metallo-β-lactamase fold. Because PqsE is an important drug target showing considerable promise for the design of new inhibitors directed against *P. aeruginosa*, mutational mapping of its active site is the first step toward exploiting functional information aimed at controlling virulence acquisition in this human pathogen.

## Materials and Methods

### Bacterial strains, plasmids, and genetic constructs

*E. coli* DH5α was grown in LB medium and on LB-agar Miller plates at 37°C. Wild-type *P. aeruginosa* strain PA14 and its *pqsE^-^* deletion mutant [10] were grown in Tryptic Soy Broth (TSB) medium (Difco) and on TSB agar plates at 37°C. Plasmid pUCP20 containing the wild-type *pqsE* gene was kindly provided by Dr. Wulf Blankenfeldt (Department of Biochemistry, University of Bayreuth, Germany) [20], [22]. The gene coding for the putative hydrolase ST1585 from the archaeon *Sulfolobus tokodaii* was codon-optimized and synthesized by GenScript. The *st1585* gene was subcloned into plasmid pUCP20 for protein expression in *P. aeruginosa*. PqsE point mutations were introduced in the pUCP20-*pqsE* plasmid using the QuikChange™ (Agilent) site-directed mutagenesis method with appropriate forward and reverse primers (Table S1 in File S1). The PCR products were chemically transformed in *E. coli* DH5α, further selected on LB-agar Miller plates with ampicillin (100 µg/mL) and verified by sequencing. The mutated plasmids were electroporated in *P. aeruginosa* PA14 *pqsE^−^* background and transformants were selected on TSB-agar plates with carbenicillin (300 µg/mL).

### Pyocyanin production and quantification

The effect of point mutations on the regulatory activity of PqsE was assessed by quantification of pyocyanin production in a *pqsE^−^* mutant of *P. aeruginosa* PA14 transformed with pUCP20-*pqsE* constructs containing the desired gene variants. A 5 mL culture of *P. aeruginosa* grown in TSB media supplemented with 100 µg/mL carbenicillin was inoculated (initial OD_600_ = 0.05) and incubated at 37°C in a rotary shaker. One mL aliquots were sampled at 3 h, 5 h, 6.5 h and 8 h to measure cell growth (OD_600_) and pyocyanin production, as previously described [23]. Briefly, 400 µL of chloroform were added to each 1-mL culture aliquots and vigorously shaken. After centrifugation, 15 µL of 0.2 M HCl were added to 300 µL of the chloroform phase. The magenta coloration of the organic phase was then quantitated at OD_520_. Concentrations, expressed as milligrams of pyocyanin produced per liter of cultured supernatant, were determined by multiplying the OD_520_ by 17.072 [23]. The experiment was performed three times. All measurements and pyocyanin quantifications were performed in triplicate to ensure reproducibility and standard deviation calculations. Low-producing variants were only considered if the average of a biological triplicate was below 50% the average of the WT PqsE enzyme production (also performed in triplicate). Similarly, drastically affected mutants were only considered if the average of a biological triplicate was below 10% that of the WT PqsE production (also performed in triplicate). These cutoff values remain very conservative, as smaller variations in pyocyanin production (<50%) are visually observable by blue pigmentation and have been shown to be biologically relevant in previous instances. These two cutoff values (10% and 50%) allowed us to identify an array of important-to-critical residues involved in maintaining the biological function of the PqsE enzyme.

## Results and Discussion

The PqsE protein from *P. aeruginosa* is encoded by the *pqsABCDE* operon, which is required for the synthesis of the small diffusible molecules AQs, including the signals HHQ (2-heptyl-4-hydroxyquinolone) and PQS (2-heptyl-3-hydroxy-4-quinolone). While the first four genes of the *pqs* operon code for enzymes involved in the biosynthesis of AQs, the last gene of this operon translates to the putative enzyme PqsE, which does not directly participate in AQ biosynthesis [10]. However, deletion of the *pqsE* gene leads to the suppression or attenuation of many virulence factors in *P. aeruginosa*, demonstrating the importance of this protein in virulence acquisition [11], [18]. Clearly, PqsE does not rely on AQs to perform its role [19]. Although the three-dimensional structure of PqsE was resolved by X-ray crystallography, wide arrays of functional assays have failed to uncover the proper molecular function of this enzyme [20]. To provide further insights into the biological function of this putative hydrolase, we first performed sequence and structural alignments of PqsE with several protein homologues and uncovered the existence of two distinct motifs potentially important for its biological function, *i.e.* two additional α-helices at the C-terminal end of the protein, and a possible hnRNP K homology domain (KH domain), which could be involved in RNA and/or ssDNA binding near the active site.

### Sequence and structure alignments with functional homologues–

**Figure 1:**
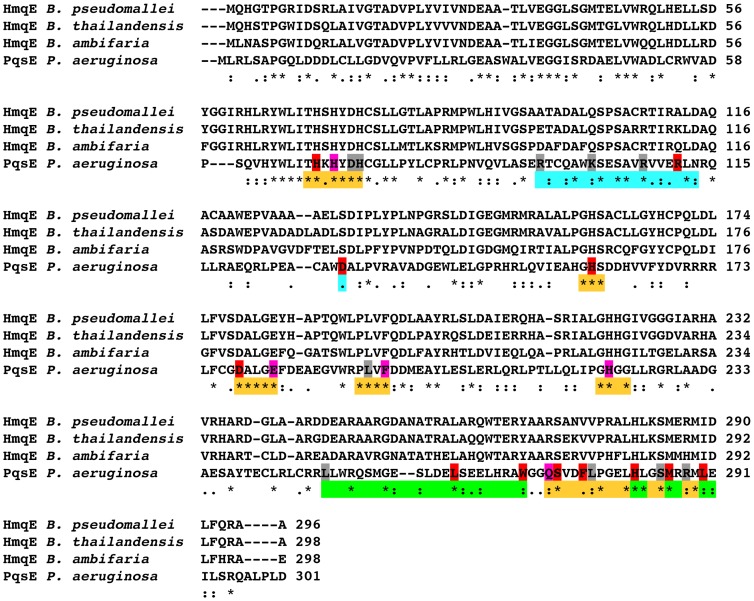
Sequence alignment of PqsE with three HmqE homologues from *Burkholderia*. Alignments were performed with ClustalW2 [26]. The asterisk (*) indicates positions which have a single, fully conserved residue; the colon (:) indicates conservation between groups of strongly similar residue properties; the period (.) indicates conservation between groups of weakly similar residue properties. Positions involved in the PqsE active site, KH-like motif and α8/α9 helices are depicted in orange, cyan, and green, respectively. PqsE residues selected for alanine scanning are colored based on their pyocyanin production in *P. aeruginosa* (see Materials and Methods): gray (no effect), magenta (moderate effect) and red (critical effect).

To date, the only enzyme known to complement the corresponding PqsE activity of a *P. aeruginosa pqsE^−^* mutant *in vivo* stems from the *pqsABCDE* homologue operon found in a few *Burkholderia* species called *hmqABCDEFG* [24], [25]. Indeed, the *hmqE* gene from *B. pseudomallei* coding for the HmqE enzyme can partially restore the pyocyanin production of a *pqsE^−^* knockout in *P. aeruginosa*, demonstrating that PqsE and HmqE play similar roles in *P. aeruginosa* and *B. pseudomallei* [24]. To identify strictly conserved residues and/or positions potentially involved in the biological function of PqsE and HmqE, we first carried out a ClustalW2 [26] sequence alignment between PqsE and the three closest *Burkholderia* HmqE homologues currently known: *B. ambifaria* (32% identity), *B. thailandensis* (31% identity), and *B. pseudomallei* (31% identity) (Figure 1). Based on this alignment, we identified a number of highly conserved residues that are most likely involved in substrate stabilization, discrimination and/or catalysis in both PqsE and HmqE. These include a total of 16 residues delineating the immediate vicinity of the active site cavity (Figure 1, orange residues), which will further be described below.

We know from the PqsE structure [20] and the SCOP database [27] that PqsE belongs to the metallo-hydrolase/oxidoreductase superfamily, a functional enzyme clan encompassing at least 13 interspecies protein families playing various biological functions. We used T-Coffee Expresso [28] to perform structure-based sequence alignments of PqsE (PDB entry 2Q0I) with 21 members of 7 metallo-hydrolase/oxidoreductase subfamilies, including zinc metallo-β-lactamases, glyoxalases, ribonuclease Z, alkyl/arylsulfatases, hydroxylases, and other putative hydrolases (Figure S1 in File S1). T-Coffee Expresso aligns protein sequences based on their 3D structure and identifies structural templates from BLAST, providing further detail on the conservation of potentially important residues in this protein fold. The aim of this structural alignment was to discriminate between residues important for folding and/or stability among the protein homologues, as opposed to residues strictly conserved for the catalytic function of the enzymes represented. The structural alignment reveals a low sequence consensus with a limited number of conserved residues throughout the sequence (Figure S1 in File S1). Conserved residues essentially localize in the vicinity of the active site environment, with no obvious conservation requirement elsewhere in the protein sequence. The only universally conserved residue among all protein homologues investigated is the catalytic D73 (PqsE numbering). Other positions showing very strong conservation (>90%) include H69, H71, H74, and H159, all of which are involved in the proper maintenance and coordination of the two active-site metal ions essential for function in this metallo-enzyme superfamily.

### Identification of two unique structural motifs in PqsE

**Figure 2:**
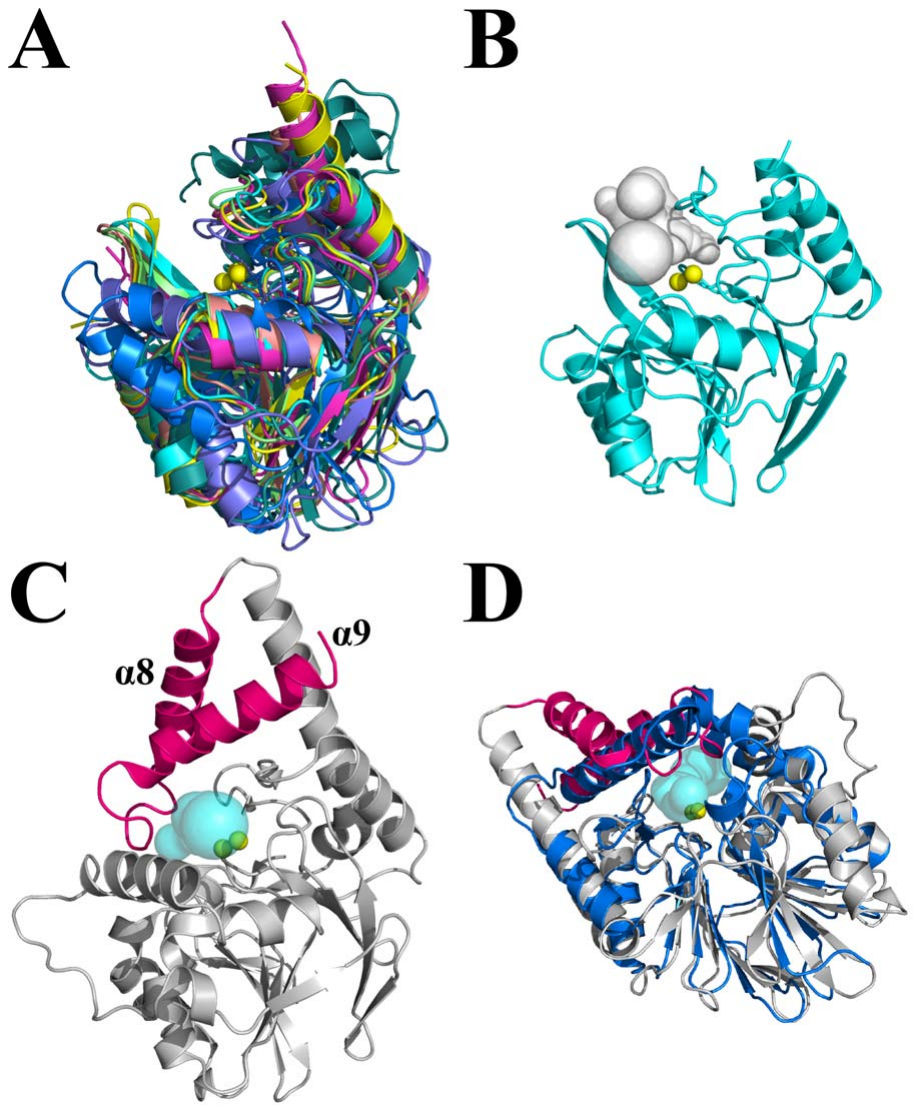
Structural overlays of PqsE homologues adopting the metallo-β-lactamase fold. A) Conserved active-site localization for eight proteins adopting the metallo-β-lactamase fold (PDB entries 1MQO, 1ZNB, 1JJT, 1KO3, 1X8H, 2YZ3, 1ZTC, and 1QH5). The two conserved active-site metal atoms are depicted as yellow spheres. B) Solvent accessible active-site cavity for a typical member of the metallo-β-lactamase fold (PDB entry 1MQO). The solvent accessible cavity was computed with CAVER [35] and is depicted as a gray surface. C) The α8 and α9 helices (in pink) restrict ligand access to the active site of PqsE by forming a narrow and elongated tunnel. The solvent accessible surface tunnel was computed with CAVER and is shown in cyan (PDB entry 2Q0I). D) Superposition of PqsE (gray) and the structural homologue ST1585 from *Sulfolobus tokodaii* (blue). ST1585 is the only structurally resolved homologue with such high similarity in the protein core (Cα RMSD of 2.8 Å) and a similar α-helical motif restricting active-site access by forming a narrow tunnel-shaped entrance (cyan surface).

PqsE adopts a very common three-dimensional structure shared by many proteins of the metallo-β-lactamase fold [29]. We retrieved all proteins adopting such a fold from the SCOP database [27], as well as members of known structure from β-lactamase families 2, B, B2, B3, and B4 (395 hits in the Pfam database [30]). The pairwise superposition of each member with PqsE was analyzed and the strong conservation among structural homologues allowed the unambiguous identification of the active-site cavity (Figure 2A). Two structural elements that differentiate PqsE from other members of this protein fold could be identified from this pairwise superposition. First, PqsE exhibits two additional α-helices (α8 and α9) at the C-terminal end of the protein (Figure 2C). These two additional helices restrict the entrance of the catalytic pocket and considerably limit solvent and substrate accessibility with respect to other members of the metallo-β-lactamase fold. While most members of this fold show an open, solvent-accessible active-site cavity access (Figure 2A–B), the two C-terminal helices of PqsE considerably narrow the active site entrance (Figure 2C). Interestingly, residues of α8 and α9 also display some of the highest B-factors of the PqsE crystal structure [22], suggesting a potential lid-like movement that could facilitate ligand binding and/or exit during catalysis. Without this concerted movement, only a small and/or elongated substrate could access the active site of PqsE through a very narrow tunnel that considerably restricts solvent and ligand accessibility to the reaction center (Figure 2C). A secondary structure prediction of the HmqE homologues also shows the presence of these additional C-terminal helices (Figure S2 in File S1) [31]. Because of their potential dynamic role in catalysis and their unique structural importance in defining the active site environment in PqsE, these two additional helices have been targeted as an interesting structural motif for further mutational and functional investigation (see below).

**Figure 3:**
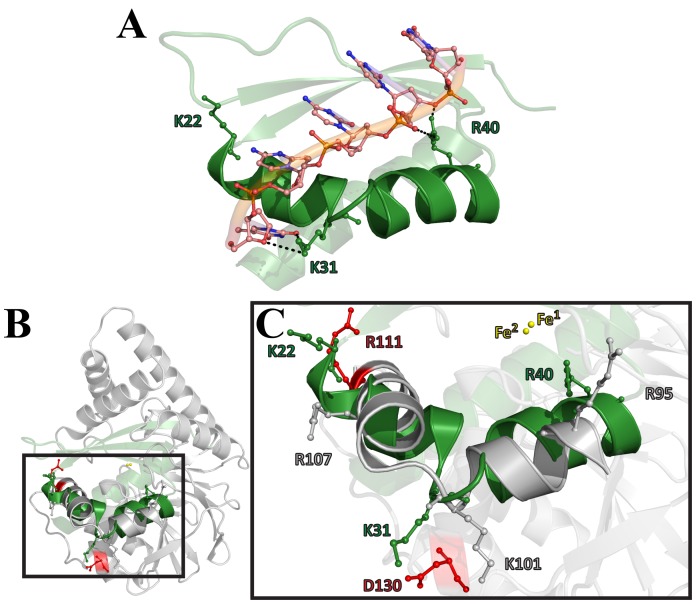
Identification of a KH-like binding motif near the active site of PqsE. KH domains are eukaryotic and prokaryotic protein motifs involved in RNA and/or ssDNA recognition found in proteins associated with transcriptional and translational regulation. A) The type I KH3 domain of hnRNP K is shown in complex with a ssDNA 10mer (PDB entry 1J5K). The positively charged residues of the KH signature (K22, K31, R40) are labeled and shown in ball-and-stick representation. B–C) Overlay of the KH3 domain of hnRNP K with the KH-like kinked helix-loop-helix motif of PqsE. Side chains of positively charged residues in the KH-like motif are labeled and displayed in ball-and-stick representation. The color code corresponds to the impact of a mutation on pyocyanin production in *P. aeruginosa* (Figure 6). The side chain of D130, which was mutated to disrupt the possible salt bridge it forms with K101, is also displayed. The two conserved active-site iron (Fe) atoms in PqsE are depicted as yellow spheres.

A previous analysis attributed a very weak phosphodiesterase activity catalyzed by PqsE on single- and double-stranded DNA and RNA substrates [20]. This result prompted us to further analyze the structural features shared between PqsE and DNA/RNase members of the metallo-β-lactamase fold. The pairwise structural alignment of PqsE with the TTHA0252 ribonuclease from *Thermus thermophilus* HB8 (PDB entry 3IE1) demonstrates that a single-stranded DNA/RNA substrate could fit in the PqsE active site tunnel, suggesting that this enzyme could be involved in RNA/DNA metabolism in *P. aeruginosa*. Additionally, the structural overlay between PqsE and TTHA0252 shows a kinked helix very similar to the helix-loop-helix motif of the hnRNP K domain (KH domain) (Figure 3). KH domains are conserved interspecies structural motifs which are typically found in proteins associated with transcriptional and translational regulation [32]. Type I KH domains are involved in RNA and/or ssDNA recognition through hydrogen bonding and electrostatic interactions between the positively charged residues on the ridge of the cleft and the negatively charged phosphate backbone of the nucleic acid [32] (Figure 3A). Interestingly, although the kinked α-helix motif of PqsE does not conserve the GXXG loop sequence typical of KH domains, it still carries positively charged residues that closely match the location of the structural equivalents involved in ssDNA and/or RNA stabilization in KH domains (Figure 3B–C). As a result, this potential KH-like motif was further considered for mutagenesis experiments in PqsE (see below).

### Mutational rationale and stability assessment

**Figure 4:**
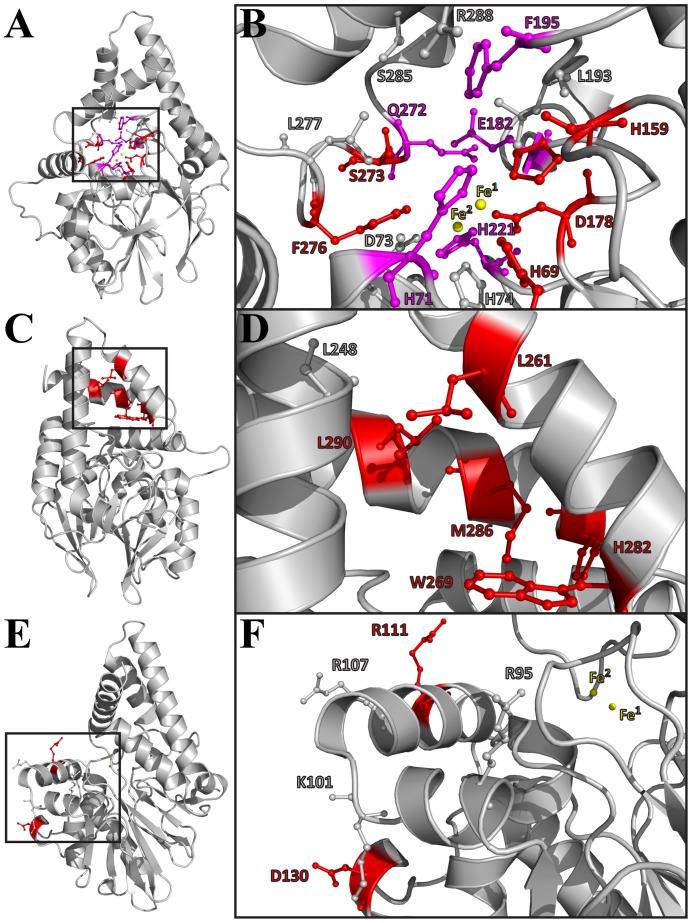
Alanine scanning of critically positioned residues in PqsE. A–B) Sixteen residues were selected based on their potential role (s) in ligand stabilization, discrimination and/or catalytic function in the vicinity of the active site cavity. C–D) Six residues were targeted in the C-terminal α8/α9 motif unique to PqsE. E–F) Five residues were selected to verify the potential importance of the KH-like motif in substrate recognition and/or stabilization. Residue side chains are labeled, displayed in ball-and-stick representation, and colored according to their impact on pyocyanin production upon mutation (Figure 6). The two catalytic iron atoms are depicted as yellow spheres.

**Figure 5:**
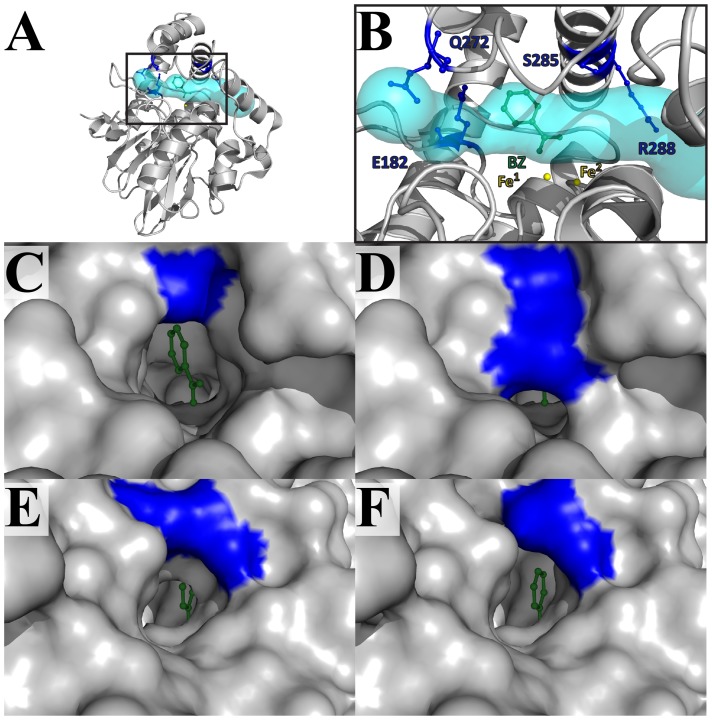
Residues delineating the tunnel-shaped active site of PqsE. A–B) Access to the active site of PqsE is restricted by a very narrow and elongated tunnel-shaped cavity displayed in cyan. The solvent accessible surface was computed with CAVER [35] and the co-crystalized benzoate moiety (BZ) is shown in green ball-and-stick representation (PDB entry 2Q0I). Residue side chains are labeled, displayed in ball-and-stick representation, and colored according to their impact on pyocyanin production upon mutation (Figure 6). C) The benzoate molecule is displayed from the S285 tunnel entrance in WT PqsE and shows clear solvent accessibility to the active site. D) The modeled S285W replacement shows the blocked entrance of the tunnel caused by the indole side chain of tryptophan. E) The benzoate molecule is displayed from the Q272 tunnel entrance in WT PqsE. F) The modeled Q272A replacement shows a modest yet significant tunnel enlargement. Amino acid replacements were generated using the PyMOL mutation function.

In light of these sequence alignments and structural analyses, a total of 27 residues were selected for mutation based on their potential role in ligand stabilization, discrimination and/or catalytic function in PqsE. Among residues located in the conserved regions identified in the first sequence alignment, 16 putative active-site positions were targeted for mutagenesis, in addition to six potentially important residues located in the unique α8/α9 C-terminal motif (Figures 1 and 4). Among the 16 active-site positions, eight were selected based on their proximity to the two coordinated iron atoms (H69, H71, D73, H74, H159, D178, H221, F276), four because of their critical location at both ends of the narrow active-site tunnel entrance (E182, Q272, S285, R288), and four were selected inside the cavity for their potential role in ligand binding (L193, F195, S273, L277) (Figure 4A–B). With the purpose of testing the importance of tunnel entrance on substrate binding and/or product release, we generated the S285W variant to restrict access to the active site (Figure 5C–D). The opposite experiment was also performed to widen the second tunnel entrance by generating the E272A variant (Fig 5E–F). The six amino acids selected in the α8/α9 motif were chosen based on their orientation, their potential to interact with a ligand in the small active-site cavity and their tendency to cluster as a hydrophobic core that maintains the two helices in place above the narrow cleft (L248, L261, W269, H282, M286, L290) (Figure 4C–D). Finally, to test the importance of the KH-like motif, we replaced each positively charged residue of the kinked α-helix motif by an alanine (R95, K101, R107, R111) to perturb stabilizing interactions normally found between KH domains and their ssDNA/RNA cognates (Figures 3 and 4E–F). We also selected D130 as a mutational target because of its potential to form a salt bridge with K101. Except for the S285W variant described above, all other positions were mutated to alanine, effectively removing the side chain functionalities found in the WT context.

While generally tolerant to subtle replacements, introducing mutations to a protein sequence can nevertheless cause significant changes in the native structure, which in turn may cause an important phenotypic change or loss of biological function. To evaluate undesired structural and/or stability changes, we used the PoPMuSiC software [21] to predict the destabilizing effects of each proposed point mutant on the PqsE structure. PoPMuSiC uses a set of 24 statistical potentials and models the volume changes caused by amino acid replacements, further expressing the folding free energy changes as a single linear combination of these terms with weighting coefficients based on solvent accessibility [21]. To identify the weighting coefficients of the energetic parameters, PoPMuSiC was trained on 2648 different point mutants from 131 unrelated proteins structures [21]. This tool evaluates the changes in stability (expressed in kcal/mol) of a given protein or peptide under single-site mutations on the basis of its resolved structure. We thus used PoPMuSiC calculations to provide an estimation of the effect of each PqsE variant on WT folding and stability, ensuring that the loss of the PqsE biological function–which translates to a loss in pyocyanin production in *P. aeruginosa* – is primarily the result of a key contact or functional interaction disruption rather than a misfolding or relative instability of the protein. Following the same rationale, we also computed the solvent accessibility (SA) of each mutated residue. Solvent accessibility is defined as the ratio of the solvent-accessible surface in the protein structure (as computed by DSSP [33]) relative to that of the same residue in an extended Gly-X-Gly tripeptide [34]. The solvent accessibility of each residue provides information about its buried state in the protein core, which was previously shown to be inversely proportional to the tolerance of a mutational replacement on protein stability [35].

Using PoPMuSiC [21], we estimated that stability changes (ΔΔ*G* ^0^) caused by mutation in the active site cavity of PqsE range from 0.1 to 2.9 kcal/mol (Table 1). Higher ΔΔ*G* ^0^ values indicate a greater propensity of the mutation to disrupt the thermodynamics of the structure, thus providing an estimation of the stability of each protein variant (Figure S3 in File S1). The most destabilizing active-site replacements predicted are H69A (2.9 kcal/mol) and F195A (2.7 kcal/mol), two variants leading to significantly lowered pyocyanin production in *P. aeruginosa* (see below). Interestingly, mutations in the α8/α9 helices are predicted to generate relatively less stable protein variants (from 1.9 kcal/mol for M286A to 3.1 kcal/mol for W269A), suggesting the importance of this unique C-terminal motif. While PoPMuSiC calculations remain predictive, we observe that pyocyanin production is typically inversely proportional to ΔΔ*G* ^0^, implying that critical active-site residues are important for both function and stability in PqsE. This prediction correlates with the very high solvent shielding propensity calculated for most active-site residues as well as for residues of the α8/α9 C-terminal motif, which generally show very low solvent accessibility ranging from 0 to 0.2 (Table 1). Indeed, all of the mutated residues in the active-site cavity are almost completely buried, except for R288 (Table 1), a side chain that points toward the exterior of the cavity. Overall, these stability predictions confirm the very hydrophobic nature of the PqsE active site and are in line with previous observations highlighting a number of instability issues linked with the expression and purification of active-site mutants [22].

**Table 1:** Pyocyanin complementation of a *P. aeruginosa* PA14 *pqsE^−^* strain transformed with the PqsE variants and the predicted free energy variation caused by each point mutation.

	PqsE Variant	Pyocyanin Production *(mg/L)* a	ΔΔ*G* ^0^ *(kcal/mol)^b^*	SA
	WT	6.2 (0.2)	–	–
Active Site [AS]	H69A	0.1 (0.1)	2.9	0.0
	H71A	2.7 (0.6)	1.5	0.2
	D73A	4.1 (0.8)	0.9	0.1
	H74A	3.5 (0.6)	2.3	0.0
	H159A	0.1 (0.0)	2.3	0.0
	D178A	0.1 (0.1)	1.4	0.0
	E182A	1.2 (0.4)	1.5	0.1
	L193A	4.7 (1.2)	2.3	0.1
	F195A	0.8 (0.3)	2.7	0.1
	H221A	1.1 (0.2)	2.3	0.1
	Q272A	1.4 (0.4)	0.9	0.2
	S273A	0.1 (0.1)	0.9	0.0
	F276A	0.1 (0.0)	2.3	0.0
	L277A	4.5 (1.1)	1.8	0.1
	S285A	5.0 (0.6)	0.3	0.1
	S285W	5.6 (0.6)	0.1	0.1
	R288A	5.2 (1.0)	0.5	0.5
C-terminal α-helix motif [α8/α9]	L248A	4.1 (1.1)	2.2	0.0
	L261A	0.2 (0.1)	2.0	0.0
	W269A	0.1 (0.0)	3.1	0.1
	H282A	0.4 (0.2)	2.0	0.1
	M286A	0.1 (0.1)	1.9	0.0
	L290A	0.1 (0.1)	2.5	0.0
KH-like motif [KH]	R95A	5.1 (0.5)	0.6	0.5
	K101A	4.4 (0.4)	0.6	0.6
	R107A	4.0 (0.8)	0.1	0.6
	R111A	0.3 (0.2)	0.2	0.6
	D130A	0.2 (0.1)	0.2	0.6

a Pyocyanin production (mg/L) was measured in triplicate as described in the experimental procedures. The mean value (± standard deviation) is shown. *^b^* Free energy variations caused by point mutants of PqsE, as predicted by the PoPMuSiC software (v.2). *^c^* Calculated solvent accessibility for each WT residue, ranging from 0 (fully buried in the protein core) to 1 (fully accessible to the solvent).

### Pyocyanin production of the KH-like motif variants

**Figure 6:**
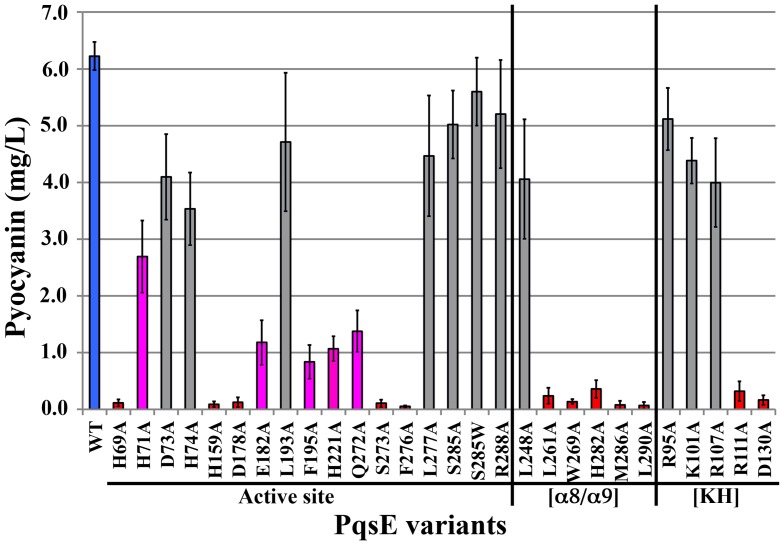
Pyocyanin production of a *P. aeruginosa* PA14 *pqsE^−^* strain transformed with WT PqsE and 28 mutational variants of PqsE. PqsE variants were generated by introducing 17 mutations in the putative active site, 6 in the additional C-terminal α8/α9 motif and 5 in the KH-like motif. Pyocyanin production (mg/L) is drastically affected for 12 PqsE variants (red, less than 10% pyocyanin production relative to WT complementation), confirming the critical role of these residues in enzyme activity and/or stability. Five low-producing variants (magenta, less than 50% pyocyanin production relative to WT complementation) still play important roles in the function of this enzyme, while 11 gray variants show no significant pyocyanin production variation relative to WT (blue).

Our pairwise superposition of PqsE with various homologues adopting the same fold suggested that the structural motif of a kinked α-helix located next to the active site could act as a ssDNA/RNA adaptor similar to that of KH domains (Figure 3). This kinked helix motif shares common features with essential components of KH domains, such as the precise positioning of positively charged residues involved in nucleic acid recognition and stabilization. In light of the fact that PqsE was previously shown to catalyze a very slow nucleolytic activity on ssDNA and RNA ligands [22], we tested whether this motif could be involved in nucleic acid recognition and/or stabilization by generating 5 critically located PqsE variants in this putative KH-like motif (Figure 4E–F). We also mutated D130 to alanine, a residue that can form a salt bridge with the positively charged K101. Our PoPMuSiC calculations for such solvent-exposed residues predicted very mild effects on the thermodynamic stability of PqsE (maximum of 0.6 kcal/mol) (Table 1). Among the five variants engineered, three do not significantly alter pyocyanin production of a *P. aeruginosa pqsE^−^* strain, suggesting that they do not play the expected role. However, PqsE variants R111A and D130A show drastically reduced pyocyanin production (Figure 6), indicating that both residues play critical roles in the molecular function of PqsE. Taking a closer look at their neighboring environment in the structure, their importance in the molecular function of PqsE remains elusive. Except for the possible salt bridge between D130 and K101, we could not identify unique interactions with neighboring partners that could justify such functional significance. Moreover, if the D130-K101 salt bridge was such an important feature of the molecular function in PqsE, removing it with the K101A mutation would have generated a similar effect on pyocyanin production, which is not observed in our analysis (Figure 6). Incidentally, R111 sits close to the entrance of the active-site tunnel and could potentially participate in ligand recognition. However, considering that D130 is a surface residue located more than 23 Å away from the closest metal ion in the active site that does not appear to make any other important link with neighboring residues, we concluded that its importance in the molecular function of PqsE is either structurally or thermodynamically defined.

### Pyocyanin production of active-site variants

The active site of PqsE forms a cavity with two octahedrally-coordinated Fe(III)/Fe(II) atoms [20], which are maintained by the same conserved sequence motif as that found in other structural homologues of the metallo-β-lactamase fold: H_69_X_aa_H_71_X_aa_D_73_H_74_∼H_159_∼H_221_ (PqsE numbering) [29]. These two PqsE active-site metal ions are 3.5 Å apart and are bridged by a water molecule and the side chain of D178 (Figure 4B). One of the iron atoms (Fe^1^) is coordinated by H69, H71, H159, and a water molecule, while the other (Fe^2^) interacts with D73, H74 and H221 (Figure 4B). Based on the position of the two iron atoms and that of small organic compounds that co-crystallized with PqsE [20], [22], we replaced the following residues to alanine in the active site pocket: H69, H71, D73, H74, H159, D178, E182, L193, F195, H221, Q272, S273, F276, L277, S285 and R288 (Figure 4A–B). Among the 17 PqsE variants with a mutated residue in the active-site environment, five are unable to complement the pyocyanin production of a *pqsE^−^* mutant of *P. aeruginosa* (H69A, H159A, D178A, S273A, F276A), five show significantly altered pyocyanin production (H71A, E182A, F195A, H221A, Q272A), and seven show minimal perturbation relative to WT PqsE (D73A, H74A, L193A, L277A, S285A, S285W, R288A) (Figure 6). These variations provide information on the relative importance of active-site positions leading to pyocyanin production in *P. aeruginosa*, and thus offer a measure of the importance of these residues in PqsE function. Much like other metallo-hydrolase/oxidoreductase homologues that rely on metal coordination for activity, the active site of PqsE is delineated by a cavity containing 5 histidine residues involved in coordinating the two metal ions, which are most likely involved in the catalytic mechanism of the enzyme (Figure 4A). While previous metal reconstitution experiments clearly identified the presence of a redox-stable Fe (III)/Fe (II) center in recombinant PqsE [20], structural homologues were previously shown to use mixed-valent metal activity centers *in vivo*. It is thus conceivable that PqsE relies on metal compositions determined by metal availability within the cell and/or at different stages of the PqsE-controlled regulation process [20]. Unexpectedly, mutating residues involved in the strictly conserved 5-histidine cluster that coordinates the two Fe atoms does not automatically result in PqsE inactivation *in vivo* (Figure 6). This observation is rather counterintuitive considering that metal ion coordination is usually critical to the function and stability of other metallo-hydrolase/oxidoreductase family members [36], [37]. However, the presence and the exact role of such metal ions are still debated [38]. While the first crystallized metallo-β-lactamase showed only one zinc atom in the active site, two-site metal binding was quickly uncovered [39], [40]. To this day, metal composition under physiological conditions remains elusive. A site-directed mutagenesis study of the metallo-β-lactamase from *Bacillus cereus* revealed that catalytic activity is reduced when mutating amino acids coordinating metal ions [41]. All mutants could bind two Zn atoms, except for one that showed increased activity with increasing zinc concentration. These observations suggest that both metal sites are required for activity, even when only one zinc atom is bound. In PqsE, replacing residues that coordinate Fe^1^ to alanine drastically affects the production of pyocyanin in *P. aeruginosa*. Indeed, variants H69A, H159A, and D178A barely allow the *in vivo* synthesis of pyocyanin while H71A significantly alters production relative to WT (Figure 6), confirming the critical importance of maintaining Fe^1^ integrity. In contrast, Fe^2^ is surrounded by residues that tolerate alanine replacements relatively well, *i.e.* D73, H74, and to a lesser extent H221. These results suggest that Fe^1^ is closely packed and more important to the catalytic mechanism of PqsE than Fe^2^.

All three experimentally determined WT structures of PqsE (PDB entries 2Q0I, 2Q0J, 2VW8) were co-crystallized in presence of small organic compounds in the active-site cavity (benzoate or cacodylate). Yu *et al.* also generated a PqsE point mutant (E182A) with an enlarged binding cavity that co-crystallized in presence of bis-*p*-nitrophenyl phosphate (bis-pNPP), a E182A-hydrolyzed substrate that is not turned over by WT PqsE [20]. Their ITC experiments also showed that a restricted set of small compounds bind to the active-site pocket of WT PqsE, namely anthranilate (*K*_D_  = 10.7±1.1 μM), benzoic acid (*K*_D_  = 29.1±4.8 μM), and synthetic benzoate derivatives such as (R)-5-bromo-2-(piperidin-3-ylamino) benzoic acid (*K*_D_  = 41±7 μM), for which the bromide moiety was shown to be critical for proper ligand binding [20], [22]. Whether all of the co-crystallized and bound ligands represent purification and/or crystallization artifacts or fortuitously bind to the pocket due to shape similarity with structural homologues remains to be determined. In fact, it is yet unknown whether these ligands really provide a clear indication of the native PqsE substrate. Our active-site mutagenesis identifies two strictly essential (S273 and F276) and three important (E182, F195 and Q272) residues contributing to function in PqsE, all of which seriously hamper or completely prevent pyocyanin production in *P. aeruginosa*. While S273 could act as the nucleophile for a typical acid/base-catalyzed reaction [20], F276 would most likely be implicated in hydrophobic and/or aromatic stacking interactions to stabilize an active-site ligand. Based on the significant level of pyocyanin production of the alanine variants, it is however doubtful that E182 and Q272 play critical roles in the enzyme mechanism, as their replacement would have otherwise completely abolished enzyme function. Globally, the fact that only 5 mutants (30%) completely abolished pyocyanin production out of the 17 replacements performed in the immediate vicinity of the two Fe atoms and co-crystallized ligands in PqsE is particularly informative. One could argue that tinkering with the immediate environment of the most important ligand cavity among structural homologues would have been more detrimental to the molecular function of PqsE. While some of these residues clearly play defining roles in substrate catalysis and/or protein stabilization, this observation suggests that the PqsE active site is in fact broader and wider than the narrow tunnel observed in the crystal structures of the enzyme, encompassing regions that are most likely exposed to ligands while the protein experiences conformational exchange. As such, our results suggest that PqsE could rely on the unique C-terminal α8/α9 motif for function, which incidentally lies right next to the active site and is very intolerant to mutation.

### Pyocyanin production of the α8/α9 motif variants

A peculiar structural motif unique to PqsE resides in the two additional α-helices that lie at the C-terminal end of the protein. While individual members of the 13 PqsE metallo-hydrolase/oxidoreductase homologues often contain additional C-terminal domains separated by unstructured linkers, PqsE is the only member displaying this unique α-helical architecture (Figure 2C). PqsE is also one of the few structural homologues where the extra structural motif is so intricately linked to the active-site environment of the protein, forming a closing lid that potentially plays a key role in capturing, releasing and/or stabilizing ligand (s) during catalysis. Since HmqE can complement the *in vivo* activity of PqsE [24] and most likely conserves the same α-helical C-terminal architecture, we generated PqsE variants by mutating six of the most conserved amino acid residues located in the α8/α9 motif (Figures 1 and 6). Five out of six replacements have a deleterious effect on pyocyanin production, confirming the critical role played by the α8/α9 motif in the molecular function of PqsE. While it is easy to envision that a residue such as H282 could play some sort of catalytic role in PqsE–especially considering how conveniently located H282 is positioned relative to the co-crystallized benzoate ligand (Fig 2D) –such rationale is not so obvious for the hydrophobic L261, M286, W269, and L290. Some of these residues are located more than 20 Å away from the two catalytic Fe atoms and could be essential for the hydrophobic packing of the α8/α9 motif, and/or for proper stabilization of a bulkier hydrophobic ligand when the lid adopts an “open” conformation. In any case, our results suggest that critical residues involved in ligand stabilization extend farther into the core of the protein, encompassing many residues of the α8/α9 motif as part of the active site of PqsE.

From structural overlays, we found that the closest non-HmqE homologue sharing a similar α-helical motif at the C-terminal end of the protein is the putative hydrolase ST1585 from the archaeon *Sulfolobus tokodaii* (PDB entry 3ADR) [42]. Crystal structures of PqsE and ST1585 overlay with a RMSD of 2.8 Å (Fig 2D), raising the possibility of a function catalyzed by the enzymatic action of ST1585 in *S. tokodaii*. To examine this possibility, we transformed a *pqsE^−^* mutant of *P. aeruginosa* with a pUCP20-*st1585* construct and quantified the resulting pyocyanin production. Expression of ST1585 in *P. aeruginosa* failed to complement the *in vivo* pyocyanin production of PqsE (not shown), suggesting that the two proteins do not act on the same regulatory system in *P. aeruginosa* and *S. tokodaii.*

## Conclusion

The present systematic mutagenesis analysis of PqsE allowed for the modulation of pyocyanin production to uncover critical residues and structural motifs affecting the catalytic activity of this enzyme required for the full virulence of *P. aeruginosa*. We reveal a critical role played by the unique C-terminal α8/α9 motif in the molecular function of PqsE, which increases the dynamic and thermodynamic complexity of the active-site cavity. Our observations lend support to a possible clamp-like motion of this motif over the active site to accommodate the PqsE substrate (s) during catalysis. Distinct mutagenesis tolerance between the two iron coordination sites also suggests a greater functional importance of the Fe^1^ ion in catalytic function. While the role and relative importance of iron atoms for the proper stability and/or biological function of PqsE remains to be assessed, our analysis suggests that Fe^2^ could be dispensable for enzyme function. Finally, mutational tolerance of the active-site cavity demonstrates high mutational robustness, an evolutionary trait pertaining to the critical role played by PqsE in virulence acquisition. Overall, our work provides molecular details for the modulation of phenazine synthesis and virulence acquisition in *P. aeruginosa* through the design of small-molecule inhibitors targeting PqsE.

## Supporting Information

File S1 **Table S1.** List of mutagenesis primers. **Figure S1**– Structure-based sequence alignment consensus between PqsE and 21 members of 7 metallo-hydrolase/oxidoreductase subfamilies and the putative hydrolase ST1585. **Figure S2**– Secondary structure prediction between PqsE and HmqE homologues. **Figure S3**– Thermodynamic stability curves for a hypothetical protein and its variant.
(DOCX)
