# Correction: Systematic Mutational Analysis of the Putative Hydrolase PqsE: Toward a Deeper Molecular Understanding of Virulence Acquisition in *Pseudomonas aeruginosa*

**DOI:** 10.1371/annotation/46f95fa7-fa55-4d4e-8c59-8aaa0c4cef04

**Published:** 2014-01-10

**Authors:** Benjamin Folch, Eric Déziel, Nicolas Doucet

An error was introduced to the second paragraph of the Introduction during production. The correct sentence is: "Interestingly, the pqsE gene is not involved in AQ production [10], [12] but still represents a key factor for the full-fledged virulence acquisition of P. aeruginosa [11], [17]."

