# Supplementary material for: Systematic Mutational Analysis of the Putative Hydrolase PqsE: Toward a Deeper Molecular Understanding of Virulence Acquisition in Pseudomonas aeruginosa
Source: PLoS One. 2013 Sep 10;8(9):e73727. doi: 10.1371/journal.pone.0073727 (PMC3769375; doi:10.1371/journal.pone.0073727)
Supplement: File S1 — Table S1. List of mutagenesis primers. Figure S1– Structure-based sequence alignment consensus between PqsE and 21 members of 7 metallo-hydrolase/oxidoreductase subfamilies and the putative hydrolase ST1585. Figure S2– Secondary structure prediction between PqsE and HmqE homologues. Figure S3– Thermodynamic stability curves for a hypothetical protein and its variant. (DOCX) [file pone.0073727.s001.docx]

**FILE S1 – SUPPORTING INFORMATION**

**Table S1.** **List of mutagenesis primers.** Only the sense-strand primers are listed (5’-3’); the anti-sense-strand primers have the same reverse-complement sequences as the corresponding sense-strand primers.

| Mutation | Mutagenesis primer sequences (5’-3’) |
| --- | --- |
| H69A | ACTACTGGCTGATCACCGCTAAGCACTACGACCACTGC |
| H71A | TGGCTGATCACCCACAAGGCTTACGACCACTGCGGCCTG |
| D73A | ATCACCCACAAGCACTACGCTCACTGCGGCCTGCTG |
| H74A | ACCCACAAGCACTACGACGCTTGCGGCCTGCTGCCCTAC |
| R95A | AGGTCCTGGCGTCCGAGGCTACCTGCCAGGCCTGG |
| K101A | ACCTGCCAGGCCTGGGCTTCGGAAAGCGCGGTG |
| R107A | AAGTCGGAAAGCGCGGTGGCTGTGGTCGAGCGCTTGAACCG |
| R111A | TGCGGGTGGTCGAGGCTTTGAACCGGCAACTGTTGCGT |
| D130A | AGGCCTGTGCCTGGGCTGCTCTGCCGGTTCGC |
| H159A | ATAGAGGCCCACGGCGCTAGCGACGATCACGTGGTTTTC |
| D178A | ACGCCTGTTCTGCGGCGCTGCCCTGGGCGAGTTCG |
| E182A | ATGCCCTGGGCGCGTTCGACGAG |
| L193A | AGAGGGGGTGTGGCGGCCGGCTGTGTTCGACGACATGGAG |
| F195A | TGGCGGCCGCTGGTGGCTGACGACATGGAGGCTTAC |
| H221A | TGCAACTGATCCCGGGAGCTGGCGGCCTGCTGCGG |
| L248A | TGTGCCGGCGGGCTCTCTGGCGCCAGTCCATG |
| L261A | AATCCCTCGACGAAGCTAGCGAGGAGCTGCACCGC |
| W269A | AGCTGCACCGCGCCGCTGGTGGGCAGAGCGTC |
| Q272A | ACCGCGCCTGGGGTGGGGCGAGCGTCGACTTCCTG |
| S273A | GGTGGGCAGGCCGTCGACTTC |
| F276A | GCAGAGCGTCGACGCTCTGCCCGGCGAACTGCACC |
| L277A | AGAGCGTCGACTTCGCACCCGGCGAACTGCACCTG |
| H282A | GCGAACTGGCCCTGGGGAGCATG |
| S285W | AACTGCACCTGGGGTGGATGCGCCGGATGCTGGAG |
| S285A | ACTGCACCTGGGGGCCATGCGCCGGATG |
| M286A | TGCACCTGGGGAGCGCTCGCCGGATGCTGGAGATTC |
| R288A | TGGGGAGCATGCGCGCGATGCTGGAGATTC |
| L290A | AGCATGCGCCGGATGGCTGAGATTCTCTCCCGCCAG |

**
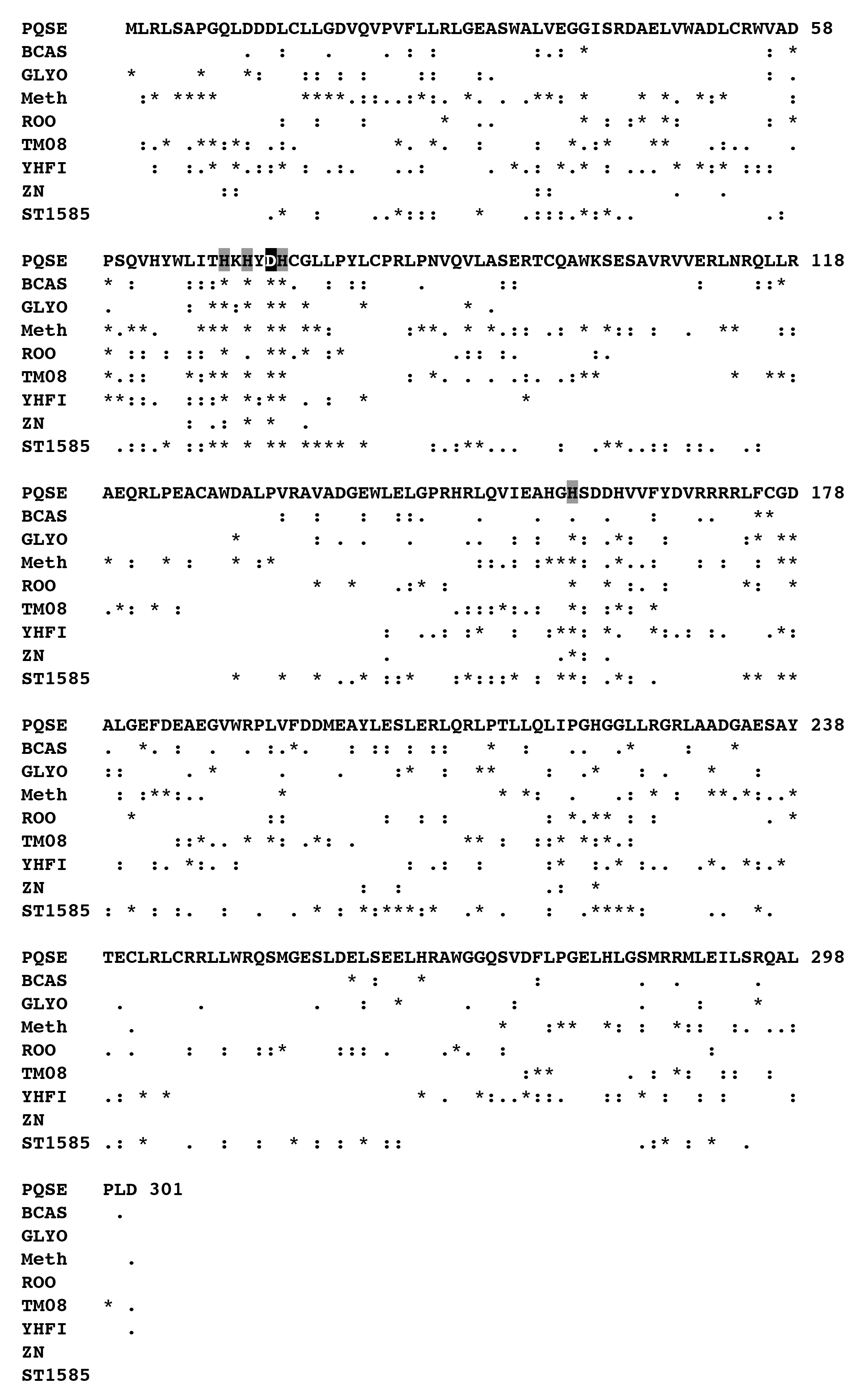
**

**Figure S1.** **Structure-based sequence alignment consensus between PqsE and 21 members of 7 metallo-hydrolase/oxidoreductase subfamilies and the putative hydrolase ST1585.** We used the T-Coffee Expresso [25] software to perform 8 individual structure-based sequence alignments of PqsE with each subfamily to retrieve their consensus sequence for fully conserved residues (*), strongly conserved residue properties (:), and weakly conserved residue properties (.). Alignments reveal that the metallo-β-lactamase fold can be achieved with a large residue composition as very few positions show conserved amino acids despite the fact that subfamily members adopt the same fold. Conserved residues essentially localize in the active site vicinity, with no obvious conservation requirement elsewhere in the protein sequence. Only the catalytic residue D73 (PqsE numbering, black box) is universally conserved among all protein homologues. Residues H69, H71, H74, and H159 (grey boxes) are also strongly conserved (>90%) and are involved in coordination of the two active-site metal ions essential for enzyme function. The protein subfamily names and the PDB codes of the different protein subfamily members are as follows: (BCAS) β-CASP RNA-metabolising hydrolases (2AZ4, 2I7T); (GLYO) Glyoxalase II hydroxyacylglutathione hydrolase (1QH5, 1XM8, 2QED); (Meth) Methyl parathion hydrolase (1P9E); (ROO) Rubreodoxyn Oxygen N-terminal domain-like (1E5D, 1VME, 1YCG); (TM08) TM0894-like (1ZTC); (YHFI) YhfI-like (1ZKP); (ZN) Zinc metallo-β-lactamase (1MQO, 1ZNB, 2AIO, 1JJT, 1KO3, 1K07, 1M2X, 1X8H, 2GMN, 2YZ3); (ST1585) ST1585 putative hydrolase from the archaeon *Sulfolobus tokodaii* (3ADR).


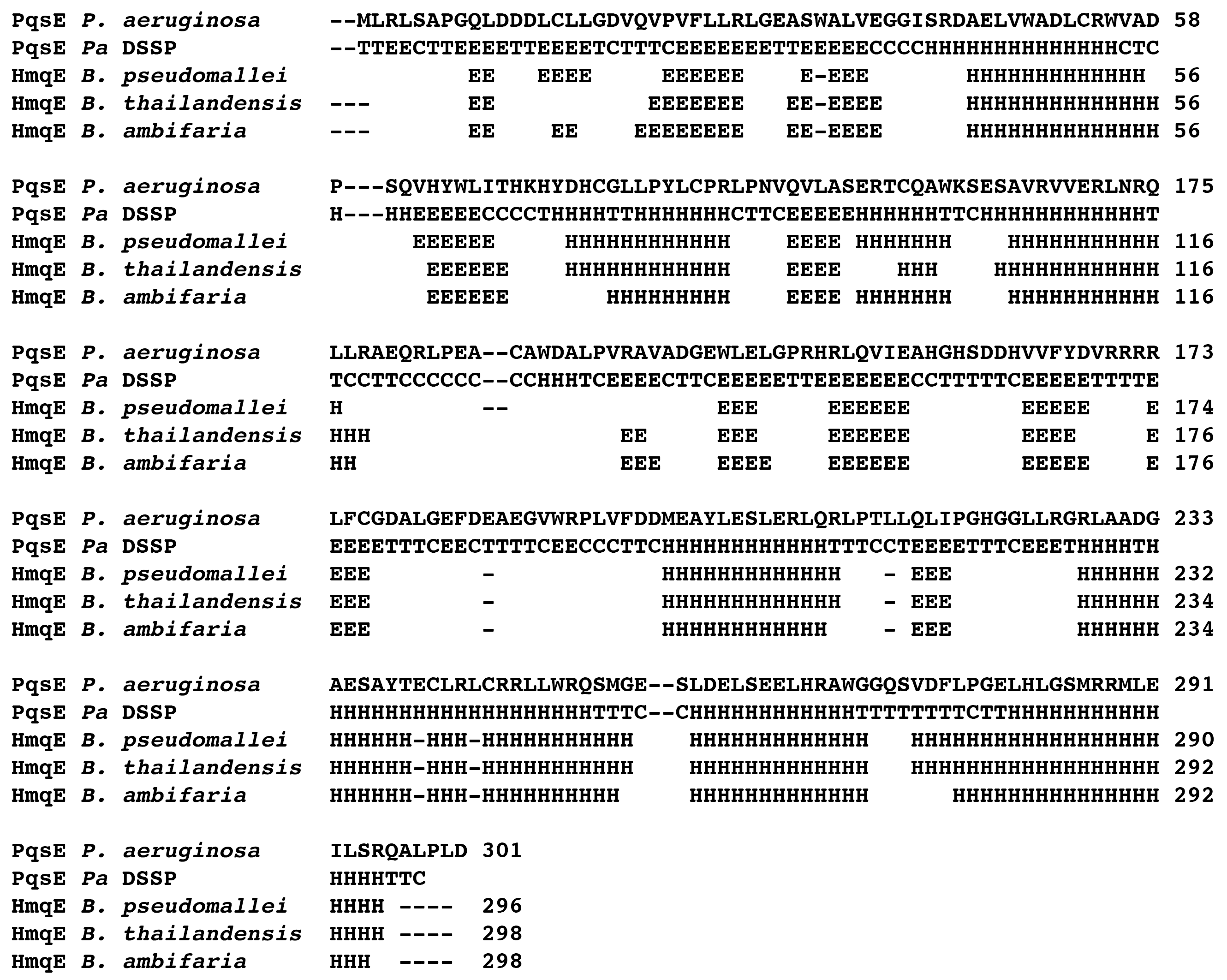


**Figure S2. Secondary structure prediction between PqsE and HmqE homologues.** Sequence alignment follows the structural alignment of Figure 1. The secondary structure of PqsE is displayed according to DSSP features, where H is used for helical motifs (G, H, and I DSSP features), E for extended motifs (E and B DSSP features), T for turn motifs (S and T DSSP features) and C for coil motifs [30]. The SYMPRED software was used to predict the secondary structure of HmqE variants [29].


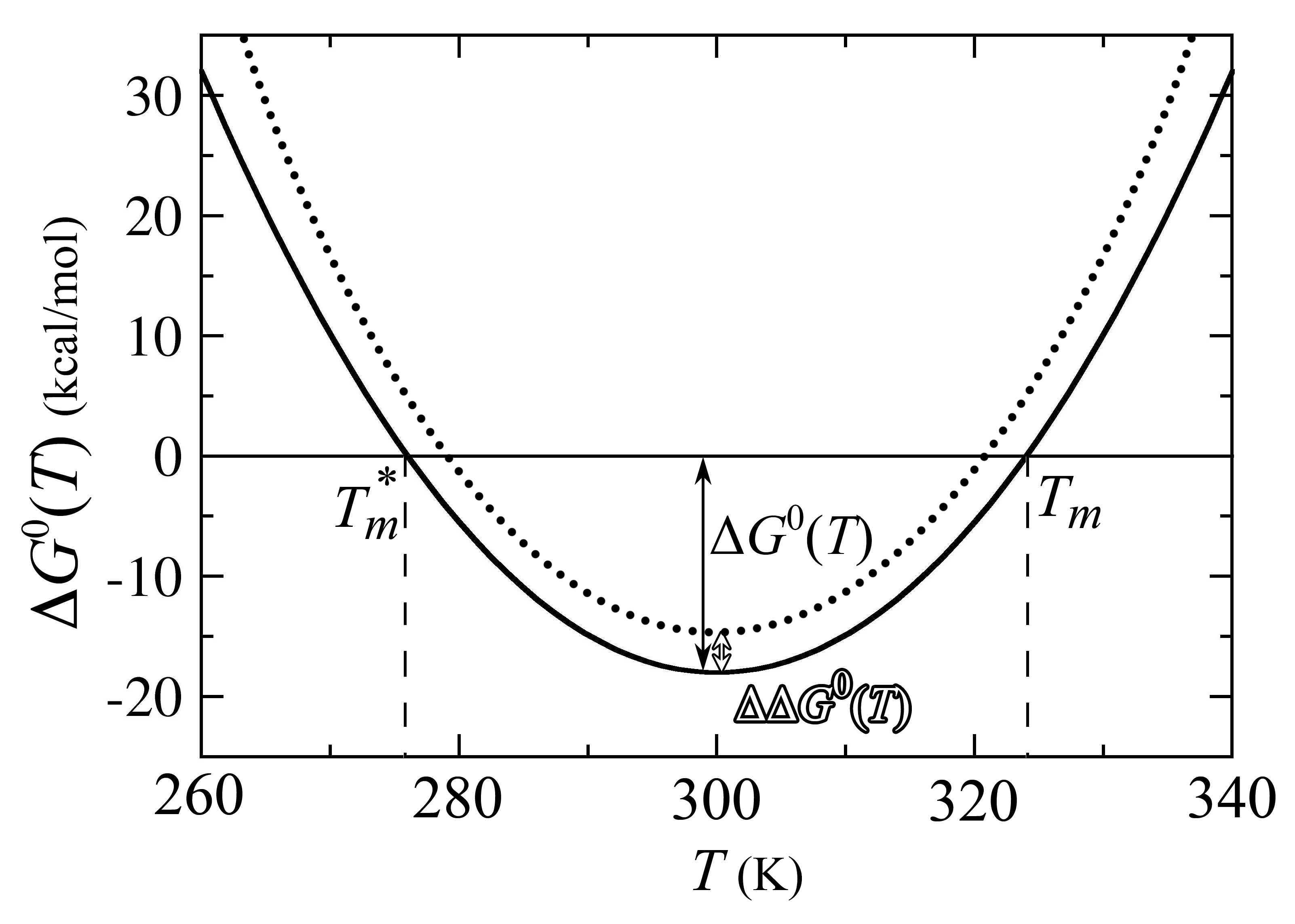


**Figure S3.** **Thermodynamic stability curves for a hypothetical protein and its variant.** The thermodynamic stability of a protein is temperature dependent and is evaluated by its folding free energy Δ*G*^0^(*T*) at room temperature (298 K). The native fold adopted by a protein is thermodynamically stable for a temperature range flanked by cold (*T_m_*^*^) and hot (*T_m_*) melting temperatures (filled line). The probability to find the protein in its native or denatured state is equal for these two melting temperatures, with a higher probability to sample the native state between the two *T_m_*s, and the unfolded state outside this temperature range. Introducing a point mutation may alter the native thermodynamic stability curve (dashed line) of the protein variant, which displays its own folding free energy Δ*G*^0^(*T*). PoPMuSiC (18) evaluates the variation of this folding free energy ΔΔ*G*^0^(*T*) to predict the thermodynamic stability changes generated by the point mutation introduced in the protein of interest.
